# Speaking the same language? A direct cartography between functional knee phenotypes and CPAK

**DOI:** 10.1002/jeo2.70801

**Published:** 2026-06-17

**Authors:** Bernard de Geofroy, Romain Léonard, Sebastien Lustig, Grégoire Micicoi

**Affiliations:** ^1^ Department of Orthopaedic Surgery Hôpital Privé Clairval, Ramsay Santé Marseille France; ^2^ Department of Orthopaedic Surgery and Traumatology Saint‐Pierre University Hospital, Centre Hospitalier Universitaire de La Réunion Saint‐Pierre France; ^3^ Orthopaedic Surgery and Sports Medicine Department FIFA Medical Center of Excellence, Croix‐Rousse Hospital, Hospices Civils de Lyon, Lyon North University Hospital Lyon France; ^4^ University Institute of Locomotor and Sports (iULS), Pasteur II Hospital Nice France; ^5^ ICARE Unit, Inserm U1091 Côte d'Azur University Nice France

**Keywords:** coronal plane alignment, CPAK, functional phenotyping, knee arthroplasty, probabilistic mapping

## Abstract

**Purpose:**

This study aimed to analytically map functional knee phenotypes (FKP) to the Coronal Plane Alignment of the Knee (CPAK) classification, evaluate CPAK's ability to represent native coronal alignment variability in non‐osteoarthritic (NO) and osteoarthritic (OA) populations and propose a simplified translational framework between both systems. It was hypothesized that CPAK represents a discretized form of native coronal alignment and that its relationship with functional phenotypes is inherently probabilistic.

**Methods:**

Arithmetic hip–knee–ankle angle and joint line obliquity were analytically derived from femoral and tibial mechanical angles, enabling direct conversion of functional phenotypes into CPAK types. A theoretical cartography was constructed using mean values and full angular intervals (±1.5°). Four assignment strategies were applied: deterministic assignment, interval propagation, stochastic Monte Carlo simulation and least probable CPAK scenario. Conversions were performed in 308 NO and 2692 OA knees from the functional phenotype cohort and compared with 500 NO and 507 OA knees from the CPAK cohort. A synthetic bidimensional framework (Hirschmann–CPAK Translational grid [HCT‐9]) was analytically derived.

**Results:**

Considering angular intervals, each functional phenotype corresponded to multiple CPAK types (mean 1.7 per phenotype), showing that deterministic conversion underestimated variability. In NO knees, distributions obtained using the mean, interval, stochastic and rare methods differed significantly from the original cohort (all *p* < 0.001). In OA knees, the distribution obtained using stochastic conversion showed no significant difference (*p* = 0.188), suggesting probabilistic convergence. Extreme types were overrepresented in the rare scenario. The HCT‐9 framework identified zones of low and high ambiguity, particularly in central phenotypic regions.

**Conclusion:**

The relationship between FKP and CPAK is inherently probabilistic. The HCT‐9 framework enables structured translation between both systems and supports a shared alignment language for total knee arthroplasty.

**Level of Evidence:**

Level III, comparative retrospective study.

## Graphical abstract

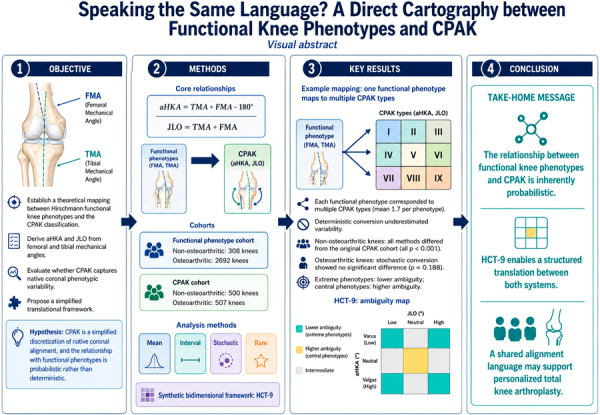

**Abbreviations**
aHKA: arithmetic hip–knee–ankle angle
CPAK: Coronal Plane Alignment of the Knee
CT: computed tomography
df: degrees of freedom
FKP: functional knee phenotypes
FMA: femoral mechanical angle
HCT‐9: Hirschmann–CPAK Translational grid
HKA: hip–knee–ankle angle
JLCA: joint line convergence angle
JLO: joint line obliquity
LDFA: lateral distal femoral angle
MPTA: medial proximal tibial angle
NO: non‐osteoarthritic
OA: osteoarthritic
OR: odds ratio
TKA: total knee arthroplasty
TMA: tibial mechanical angle
*χ* ^2^
: chi‐square test
Δ*χ* ^2^
: change in chi‐square statistic

## INTRODUCTION

Understanding native coronal knee alignment has become a major focus in the development of personalized alignment strategies in total knee arthroplasty. For several decades, mechanical alignment was considered the gold standard. However, substantial evidence derived from cohorts of young non‐arthritic individuals, as well as patients with knee osteoarthritis, has demonstrated considerable interindividual variability in coronal knee alignment, thereby challenging the universality of neutral mechanical alignment [17]. Beyond the global lower limb axis, several studies have shown that coronal knee alignment results from the combination of independent osseous parameters, particularly distal femoral orientation and proximal tibial orientation. The femoral mechanical angle (FMA or lateral distal femoral angle [LDFA]) and the tibial mechanical angle (TMA or medial proximal tibial angle [MPTA]) exhibit a wide and continuous distribution in both non‐arthritic populations and patients with knee osteoarthritis [4, 6, 9, 10]. Consequently, markedly different femorotibial configurations may lead to an identical hip–knee–ankle (HKA) angle, highlighting the limitations of a unidimensional approach to coronal alignment.

In this context, Hirschmann et al. introduced the concept of functional knee phenotypes (FKP), based on the combination of global lower limb alignment and the orientation of the femoral and tibial articular surfaces [9]. This classification, developed from three‐dimensional computed tomography (CT) imaging in young non‐arthritic subjects, describes coronal alignment as a continuum of phenotypes defined by angular ranges around mean values. Subsequent work by the same group confirmed the substantial variability of femoral and tibial phenotypes, as well as their non‐Gaussian distribution within the general population [4]. More recently, MacDessi et al. proposed the Coronal Plane Alignment of the Knee classification (CPAK), based on two geometrically independent parameters: the arithmetic hip–knee–ankle angle (aHKA), representing constitutional lower limb alignment and joint line obliquity (JLO), defined as the sum of femoral and tibial orientations [16]. By combining these two dimensions, the CPAK system defines nine discrete phenotypes and provides a standardized and pragmatic framework for describing coronal alignment. However, although CPAK offers the advantages of clarity and ease of clinical use, it relies on the discretization of inherently continuous parameters and does not explicitly account for the interval‐based nature of native anatomical phenotypes. In contrast, the functional classification proposed by Hirschmann, which is more descriptive and grounded in angular ranges, more precisely captures the intrinsic variability of coronal alignment, albeit at the expense of greater complexity and more limited clinical applicability. To date, no study has yet formally analysed the geometric relationships between these two classification systems or evaluated the extent to which CPAK can reproduce the phenotypic diversity described by the functional approach of Hirschmann et al.

The present study aimed to establish a theoretical mapping between the FKP described by Hirschmann and the CPAK classification by analytically deriving the arithmetic HKA angle and JLO from FMA and TMA. This mapping was then applied to population‐based data to determine whether the CPAK system adequately captures native phenotypic variability of the knee. Finally, a simplified interpretative framework was proposed to bridge both classification systems. It was hypothesized that CPAK represents a simplified discretization of native coronal alignment and that the relationship between FKP and CPAK types is intrinsically probabilistic rather than deterministic.

## MATERIALS AND METHODS

### Geometric definitions

#### Definitions of the CPAK classification

**Figure 1:**
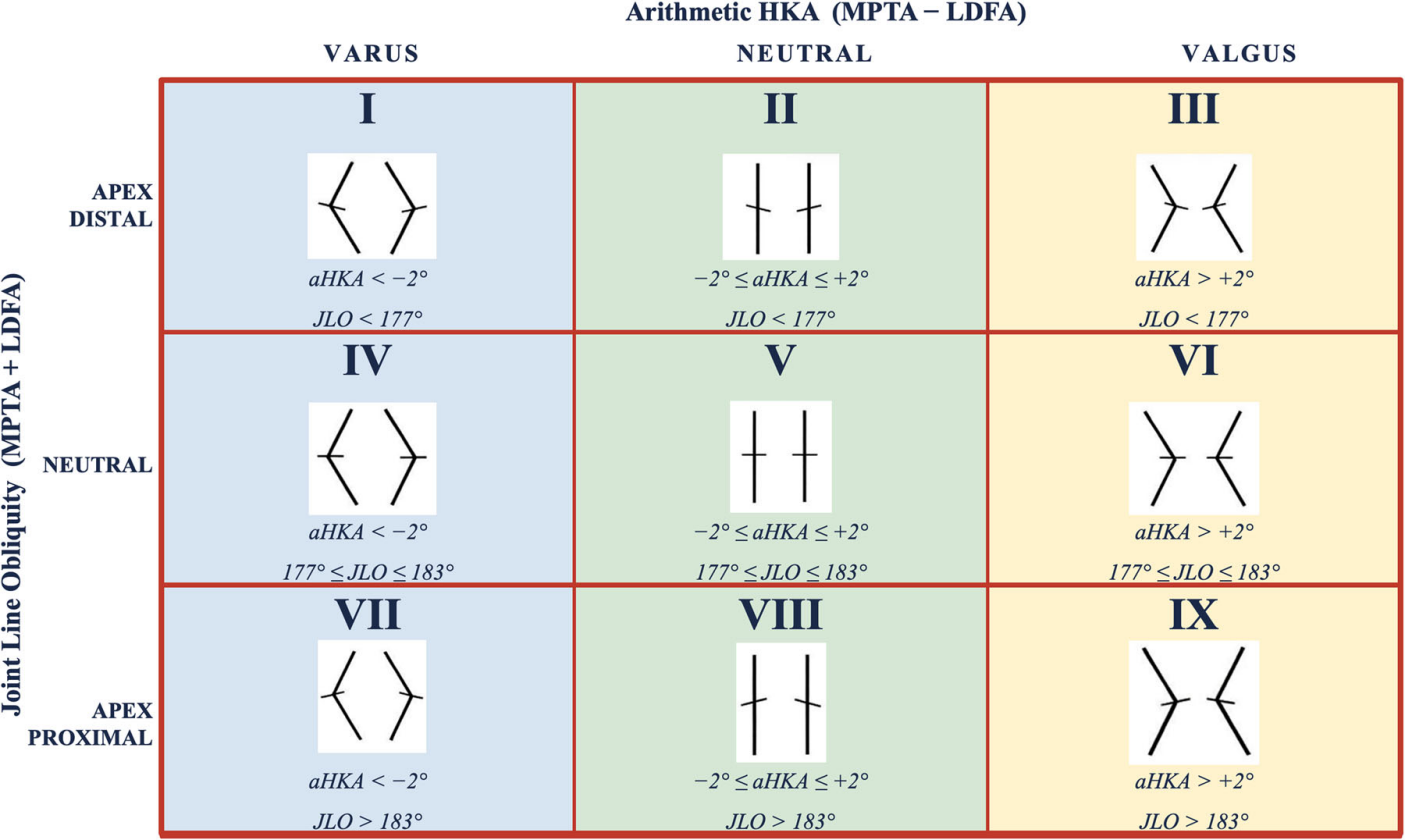
Coronal Plane Alignment of the Knee (CPAK) classification with nine theoretical knee types. *Source*: Adapted from MacDessi et al. [16]. aHKA, arithmetic hip–knee–ankle angle; HKA, hip–knee–ankle angle; JLO, joint line obliquity; LDFA, lateral distal femoral angle; MPTA, medial proximal tibial angle.

The CPAK classification, described by MacDessi et al. [16], is based on two geometrically independent parameters derived from the FMA and TMA measured on full‐length weight‐bearing radiographs. The aHKA represents the constitutional alignment of the lower limb, independently of JLO (Figure 1). It is defined as the arithmetic difference between the MPTA and the LDFA:

aHKA=MPTA−LDFA.

It should be noted that the arithmetic aHKA differs from the measured HKA obtained on weight‐bearing radiographs, which additionally incorporates the joint line convergence angle (JLCA). Throughout this study, aHKA is used exclusively in its arithmetic definition, consistent with the CPAK framework as originally described by MacDessi et al. A negative aHKA corresponds to constitutional varus alignment, whereas a positive aHKA indicates constitutional valgus alignment. The second parameter is JLO, which reflects the combined orientation of the femoral and tibial articular surfaces relative to the horizontal plane. It is defined as the sum of the tibial and FMAs:

JLO=MPTA+LDFA.

According to MacDessi et al., a JLO lower than 177° corresponds to an apex distal joint line, a JLO between 177° and 183° to a neutral joint line and a JLO greater than 183° to an apex proximal joint line. The combination of aHKA categories (varus, neutral, valgus) and JLO categories (apex distal, neutral, apex proximal) defines the nine CPAK phenotypes.

#### Definitions of the FKP

The FKP described by Hirschmann et al. [11] are based on the analysis of coronal alignment derived from three‐dimensional CT imaging performed in the supine position in young non‐osteoarthritic (NO) subjects. This classification was designed to characterize native coronal knee alignment while accounting for individual anatomical variability, rather than relying on a theoretical geometric neutral reference. Within this framework, mechanical angles are measured medially and phenotypic neutrality is not defined by a fixed angular value of 90°, but by population‐based mean values derived from the Hirschmann reference cohort. These reference values are cohort‐specific and may differ across populations with distinct demographic or racial characteristics. The corresponding mean values are 93° for the FMA and 87° for the TMA. The FMA is defined as the angle formed between the mechanical axis of the femur and the distal femoral articular surface, measured medially. An FMA value lower than the population mean (93°) corresponds to a relatively varus‐oriented femur, whereas a greater value corresponds to a relatively valgus‐oriented femur. Similarly, the TMA is defined as the angle between the mechanical axis of the tibia and the proximal tibial articular surface, measured medially. A TMA value lower than the population means (87°) corresponds to a relatively varus‐oriented tibia, whereas a greater value corresponds to a relatively valgus‐oriented tibia. FKP are therefore defined as angular intervals centred around these mean values, in 3° increments (±1.5° around each central value), rather than as single‐point measurements. This interval‐based approach allows coronal knee alignment to be described as a continuum of femorotibial configurations and more accurately reflects the native anatomical variability observed in the NO population.

#### Geometric conversions

To establish a direct geometric mapping between FKP and the CPAK classification, the CPAK parameters (aHKA and JLO) must be expressed as functions of the FMA and TMA angles defined by Hirschmann. Since the FMA is measured medially in the Hirschmann classification, whereas the LDFA is measured laterally in the CPAK framework, the following geometric relationship applies:

$$\mathrm{LDFA}=180^{\circ }-\mathrm{FMA}.$$

In contrast, the TMA, measured medially, is geometrically equivalent to the MPTA used in the CPAK classification:

MPTA=TMA.

By substituting these relationships into the equations defined by MacDessi et al., the CPAK parameters can be directly expressed as functions of the Hirschmann femoral and tibial angles:

$$\mathrm{aHKA}=\mathrm{TMA}+\mathrm{FMA}-180^{\circ },$$

$$\mathrm{JLO}=180^{\circ }+(\mathrm{TMA}-\mathrm{FMA}).$$

These equations demonstrate that the CPAK classification is entirely determined by the (FMA, TMA) pair, independently of a direct HKA measurement. Consequently, each functional phenotype, defined as an interval of FMA and TMA values, does not correspond to a single CPAK type, but rather to a range of possible aHKA and JLO values, and therefore potentially to multiple distinct CPAK phenotypes.

### Theoretical mapping

FKP were mapped using two complementary approaches. The first mapping was performed using the mean FMA and TMA values defining each functional phenotype, allowing assignment to a single deterministic CPAK type. The second mapping incorporated the full angular intervals (±1.5°) of FMA and TMA, thereby reflecting native anatomical variability. Propagation of these intervals into the CPAK parameters (aHKA and JLO) generated continuous ranges of values, leading to overlapping CPAK categories when a single functional phenotype corresponded to multiple possible CPAK types.

### Study populations

Population data derived from the study by Hirschmann et al., including NO knees (308 knees) and osteoarthritic (OA) patients (2692 knees) assessed using standardized three‐dimensional CT protocols, were analysed. Inclusion and exclusion criteria were those reported in the original studies, notably excluding patients with a history of prior surgery, fracture or major deformity. The resulting distributions were subsequently compared with those reported in the original study by MacDessi et al. (500 NO knees and 507 OA knees) to evaluate population‐level concordance between functional phenotypes and the CPAK classification.

### CPAK attribution

CPAK phenotypes were assigned to the Hirschmann population using four methodological approaches. The ‘mean’ method relied on the exact central FMA and TMA values defining each functional phenotype, allowing deterministic assignment to a single CPAK category. The ‘interval’ method considered the full angular ranges of FMA and TMA (±1.5°), with analytical propagation into the aHKA–JLO space to identify all CPAK types theoretically compatible with a given functional phenotype. A ‘stochastic’ Monte Carlo approach was subsequently applied, simulating a realistic patient distribution by randomly generating FMA–TMA pairs within each interval assuming a centred Gaussian distribution, thereby estimating the probability of belonging to each CPAK type. Finally, the ‘least probable CPAK’ method identified, among all compatible CPAK types, those corresponding to the marginal regions of the aHKA–JLO space, referred to as ‘rare’ CPAK types. Results from these different approaches were aggregated at the population level to analyse and compare overall CPAK distributions according to the attribution strategy. To ensure adequate cell counts and limit low‐frequency categories, CPAK phenotypes were grouped into four clinically coherent clusters (I–II, III–IV, V and ≥VI). This grouping was motivated by the predominance of Types I and II and the relative rarity of Types VII to IX.

### Hirschmann‐CPAK translational grid (HCT‐9)

The HCT‐9 grid partitions the space of Hirschmann functional phenotypes (FMA, TMA) into a 3 × 3 matrix ranging from A1 to C3 according to CPAK‐derived thresholds applied to the anatomical axes. The horizontal axis represents the femoral phenotype (FMA < 91.5° = varus; 91.5°–94.5° = neutral; >94.5° = valgus). The vertical axis represents the tibial phenotype (TMA < 85.5° = varus; 85.5°–88.5° = neutral; >88.5° = valgus). For each cell, the set of possible CPAK types is determined through analytical transformation using the equations aHKA = TMA + FMA − 180° and JLO = TMA + FMA, followed by application of CPAK thresholds (aHKA: ±3°; JLO: 177°–183°). This approach allows identification of regions with low ambiguity (1–2 possible CPAK types per cell) and high ambiguity (4–6 possible types).

### Statistical analysis

CPAK phenotype distributions were described using absolute and relative frequencies after grouping the nine CPAK types into four categories (I–II, III–IV, V and ≥ VI) to avoid low cell counts and ensure the validity of statistical tests. As only aggregated data were available, comparisons between classifications, attribution methods and populations were performed using unpaired chi‐square (*χ* ^2^) tests applied to contingency tables. CPAK distributions were compared separately according to osteoarthritis status (NO vs. OA) and attribution method (mean, interval, stochastic and rare). *p* values were adjusted for multiple comparisons using the Holm–Bonferroni method, with statistical significance set at *p* < 0.05 after correction. An exploratory binary logistic regression analysis was conducted to assess whether the relative proportions of NO and OA knees predicted cohort membership (Hirschmann vs. MacDessi). All analyses were performed using JASP software (Version 0.95.1).

## RESULTS

### Theoretical mapping

**Figure 2:**
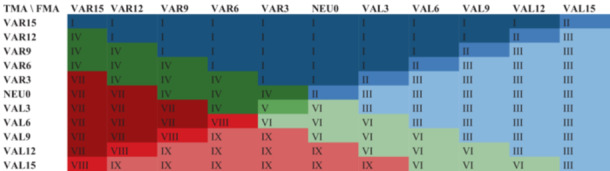
Deterministic mapping between functional knee phenotypes (FMA mean = *x*‐coordinate − TMA mean = *y*‐coordinate) and CPAK classification femoral. The *x*‐axis represents the femoral mechanical angle (FMA): values to the left (FMA < 93°) correspond to varus femoral orientation, values to the right (FMA > 93°) to valgus femoral orientation. The *y*‐axis represents the tibial mechanical angle (TMA): values below (TMA < 87°) correspond to varus tibial orientation, values above (TMA > 87°) to valgus tibial orientation. Population means (FMA = 93°, TMA = 87°) serve as phenotypic neutrality references per the Hirschmann classification. CPAK, Coronal Plane Alignment of the Knee.

**Figure 3:**
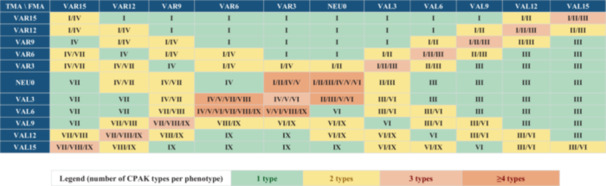
Correspondence between functional knee phenotypes and CPAK types across femoral (FMA = *x*‐coordinate) and tibial (TMA = *y*‐coordinate) mechanical angle intervals. Each functional phenotype corresponds to a 3° angular interval, defined as ± 1.5° around the central femoral (FMA) and tibial (TMA) mechanical angle values. The table illustrates the theoretical mapping between these FMA–TMA intervals and the CPAK classification. When the angular interval of a phenotype intersects several CPAK regions, multiple CPAK types are listed within the same cell. CPAK, Coronal Plane Alignment of the Knee.

Figure 2 illustrates the deterministic correspondence between FKP, defined by the mean FMA and TMA values and CPAK classification types. Combined femoral and tibial varus configurations are predominantly associated with CPAK Types I and IV, whereas near‐neutral alignments correspond mainly to types II and III. Conversely, increasing combined valgus alignment is associated with CPAK Types VI–IX. This mapping demonstrates a progressive and coherent organization of CPAK types according to the mean combined coronal alignment of the femur and tibia. Appendix S1 provides a detailed correspondence between FKP and CPAK classification based on mean mechanical axis values. However, this deterministic approach underestimates variability and does not account for intra‐phenotype dispersion. When strictly applying the definition of FKP based on FMA and TMA intervals (±1.5°), the correspondence between the two classifications involves multiple CPAK types for a single FKP category. On average, one FKP corresponds to 1.7 CPAK types (Appendices S2 and S3). Figure 3 shows a progressive organization of CPAK types according to the combined femoral (FMA) and tibial (TMA) mechanical axis intervals, with unambiguous correspondences primarily observed at the varus and valgus extremes. In contrast, central regions are associated with multiple CPAK types, reflecting transitional functional phenotypes.

### Comparison according to attribution method and osteoarthritis status

#### ‘Mean’ method

**Figure 4:**
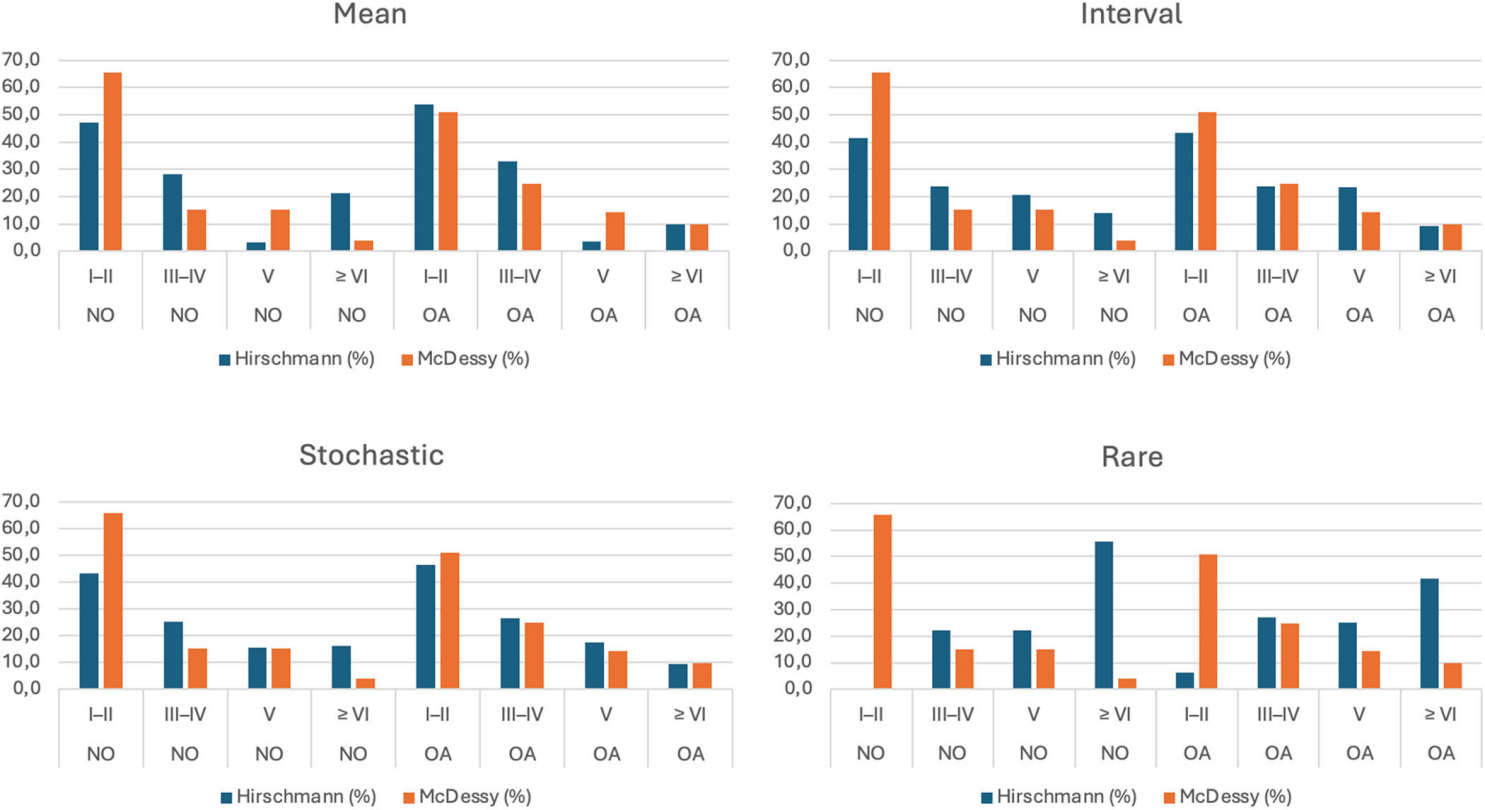
Distribution of functional knee phenotypes according to the Hirschmann and MacDessi (CPAK) classifications in osteoarthritic (OA) and non‐osteoarthritic (NO) populations. CPAK distributions obtained from the Hirschmann functional phenotype cohort are compared with those reported in the original CPAK cohort (MacDessi) in NO and OA populations. Four attribution strategies were applied: mean, using the central FMA and TMA values; interval, considering the full angular ranges (±1.5°) of each phenotype; stochastic, using Monte Carlo sampling within these intervals; and rare, selecting CPAK types located at the margins of the compatible aHKA–JLO space. aHKA, arithmetic hip–knee–ankle angle; CPAK, Coronal Plane Alignment of the Knee; FMA, femoral mechanical angle; JLO, joint line obliquity; TMA, total knee arthroplasty.

Using deterministic conversion based on mean values, marked differences were observed between the NO populations of Hirschmann and MacDessi. In the Hirschmann cohort, CPAK Types I–II accounted for 47.1% of cases, compared with 65.6% in the MacDessi cohort, whereas Types ≥ VI were substantially more frequent in the Hirschmann population (21.4% vs. 4.0%). The proportion of CPAK Type V was significantly lower in the Hirschmann cohort (3.3%) than in the MacDessi cohort (15.2%). Overall, the distribution of CPAK categories differed significantly between the two cohorts (*χ* ^2 ^= 107.2; degrees of freedom [df] = 3; *p* < 0.001; total *N* = 808). Similar differences were observed in the OA population. Although the proportions of extreme phenotypes (≥VI) were nearly identical between the Hirschmann and MacDessi cohorts (9.9% vs. 9.9%), CPAK Type V was markedly underrepresented in the Hirschmann‐derived distribution (3.6%) compared with the MacDessi cohort (14.4%). The overall CPAK distribution remained significantly different between the two cohorts (*χ* ^2 ^= 104.0; df = 3; *p* < 0.001; total *N* = 3199) (Figure 4).

#### ‘Interval’ method

When the full FMA and TMA intervals were considered, the CPAK distribution derived from the Hirschmann cohort became more dispersed. Among NO knees, Types I–II accounted for 41.5% of cases in the Hirschmann population compared with 65.6% in the MacDessi cohort, whereas Type V increased to 20.8% versus 15.2%, respectively. Despite this redistribution, the overall distributions remained significantly different (*χ* ^2 ^= 79.3; df = 3; *p* < 0.001; total *N* = 1242). In the OA population, the distributions obtained using the interval method were more comparable between cohorts. Nevertheless, statistically significant differences persisted (*χ* ^2 ^= 23.1; df = 3; *p* < 0.001; total *N* = 6694), indicating incomplete convergence between the Hirschmann‐derived CPAK distributions and those originally reported by MacDessi.

#### ‘Stochastic’ method

With stochastic sampling within the FMA–TMA intervals, the NO Hirschmann cohort continued to exhibit a higher proportion of extreme phenotypes compared with MacDessi, particularly for Types ≥ VI (16.2% vs. 4.0%). The overall CPAK distribution differed significantly between the two cohorts (*χ* ^2 ^= 73.3; df = 3; *p* < 0.001; total *N* = 1026). In contrast, no statistically significant difference was observed between the Hirschmann‐derived and MacDessi CPAK distributions in the OA population when the stochastic method was applied (*χ* ^2 ^= 4.79; df = 3; *p* = 0.188; total *N* = 4945). The relative proportions across all CPAK strata (I–II, III–IV, V and ≥ VI) were comparable between the two cohorts.

#### ‘Rare’ method

The ‘rare’ CPAK scenario resulted in markedly asymmetric distributions in the NO Hirschmann cohort, with no cases classified as I–II and more than half of knees categorized as ≥VI (55.6%). In contrast, the MacDessi cohort showed a distribution predominantly composed of lower‐grade CPAK types. The difference between the two cohorts was highly significant (*χ* ^2 ^= 92.7; df = 3; *p* < 0.001; total *N* = 518). Among OA knees, the ‘rare’ method similarly overrepresented extreme CPAK phenotypes in the Hirschmann cohort (Types ≥ VI: 41.7%) compared with MacDessi (9.9%). The resulting distributions differed significantly (*χ* ^2 ^= 57.1; df = 3; *p* < 0.001; total *N* = 555).

### Additional factors influencing CPAK distribution

Regardless of the attribution method used, the presence of osteoarthritis significantly influenced CPAK distribution. Using the ‘mean’ method, CPAK distributions differed significantly between NO and OA knees (*χ* ^2 ^= 61.4; df = 3; *p* < 0.001; *N* = 4007). Similar significant differences were observed with the interval method (*χ* ^2 ^= 26.5; *p* < 0.001; *N* = 7936), the stochastic method (*χ* ^2 ^= 23.3; *p* < 0.001; *N* = 5971) and the rare method (*χ* ^2 ^= 38.4; *p* < 0.001; *N* = 1073). When NO and OA knees were analysed jointly, significant differences were consistently observed between the CPAK distributions derived from Hirschmann and those reported by MacDessi. This effect was found with the ‘mean’ method (*χ* ^2 ^= 205.7; df = 3; *p* < 0.001; *N* = 4007), the stochastic method (*χ* ^2 ^= 50.3; *p* < 0.001; *N* = 5971) and the ‘rare’ method (*χ* ^2 ^= 135.6; *p* < 0.001; *N* = 1073) (Table 1). A binary logistic regression analysis was conducted to assess whether the relative proportions of NO and OA knees predicted cohort membership (Hirschmann vs. MacDessi) under the ‘mean’ method. The overall model significantly improved fit compared with the null model (Δ*χ* ^2 ^= 16.4; *p* < 0.001), with high pseudo‐*R* ^2^ values. However, neither the number of NO knees (odds ratio [OR] = 1.66; *p* = 0.310) nor the number of OA knees (OR = 0.87; *p* = 0.276) emerged as independent predictors of cohort membership.

**Table 1:** Influence of osteoarthritis status (OA vs. NO knees) and population on CPAK distributions according to attribution method.

Method	Comparison	*χ* ^2^	df	*p*	*N*	Cramér's *V*
Mean	NO versus OA	61.44	3	<0.001	4007	0.071
	Hirschmann versus MacDessi (global)	205.7	3	<0.001	4007	0.131
	Hirschmann versus MacDessi (NO)	107.2	3	<0.001	808	0.210
	Hirschmann versus MacDessi (OA)	104.0	3	<0.001	3199	0.104
Interval	NO versus OA	26.51	3	<0.001	7936	0.033
	Hirschmann versus MacDessi (NO)	79.27	3	<0.001	1242	0.146
	Hirschmann versus MacDessi (OA)	23.11	3	<0.001	6694	0.034
Stochastic	NO versus OA	23.31	3	<0.001	5971	0.036
	Hirschmann versus MacDessi (global)	50.34	3	<0.001	5971	0.053
	Hirschmann versus MacDessi (NO)	73.31	3	<0.001	1026	0.155
	Hirschmann versus MacDessi (OA)	4.79	3	0.188	4945	0.018
Rare	NO versus OA	38.44	3	<0.001	1073	0.109
	Hirschmann versus MacDessi (global)	135.6	3	<0.001	1073	0.205
	Hirschmann versus MacDessi (NO)	92.66	3	<0.001	518	0.244
	Hirschmann versus MacDessi (OA)	57.05	3	<0.001	555	0.185

*Note*: Effect sizes were generally small to moderate (Cramér's *V* ranging from 0.018 to 0.244). The only non‐significant comparison was observed for the stochastic method in OA knees (*χ* ^2 ^= 4.79; *p* = 0.188; *V* = 0.018), indicating near‐complete probabilistic convergence between cohorts.

Abbreviations: aHKA, arithmetic hip–knee–ankle angle; CPAK, Coronal Plane Alignment of the Knee; df, degrees of freedom; FMA, femoral mechanical angle; HKA, hip–knee–ankle angle; JLO, joint line obliquity; LDFA, lateral distal femoral angle; MPTA, medial proximal tibial angle; NO, non‐osteoarthritic; OA, osteoarthritic; TMA, tibial mechanical angle; *χ* ^2^, chi‐square statistic.

### Hirschmann–CPAK translational grid (HCT‐9)

**Figure 5:**
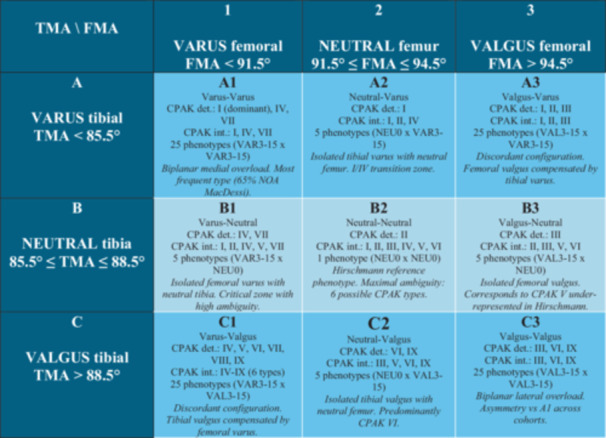
Hirschmann‐CPAK (HCT‐9) reading grid. CPAK, Coronal Plane Alignment of the Knee; FMA, femoral mechanical angle; HCT‐9, Hirschmann–CPAK Translational grid; TMA, tibial mechanical angle.

Figure 5 highlights a structured and progressive organization of the correspondence between FKP and CPAK types along two independent axes: the constitutional femoral phenotype and the articular tibial phenotype. Extreme biplanar configurations (A1 varus–varus and C3 valgus–valgus) demonstrate a relatively unambiguous correspondence with CPAK, whereas central and discordant configurations are associated with a marked increase in the number of compatible CPAK types. Table 2 further substantiates this hierarchy by quantifying the degree of conversion ambiguity for each HCT‐9 cell. The B2 cell (neutral–neutral), which represents Hirschmann's reference phenotype, exhibits maximal ambiguity, with six possible CPAK types. Adjacent cells (B1, B3, C1 and C2) also display high levels of ambiguity, whereas only peripheral cells allow for reliable deterministic assignment. Collectively, these findings demonstrate that the Hirschmann‐CPAK relationship is fundamentally probabilistic. The HCT‐9 framework enables precise identification of regions in which a stochastic approach is required to avoid reductive classification during Hirschmann‐to‐CPAK conversion. The choice between deterministic and stochastic attribution is therefore not arbitrary but directly dictated by the geometric ambiguity inherent to the Hirschmann‐CPAK projection. When the angular interval of a given Hirschmann phenotype intersects multiple CPAK regions, deterministic assignment becomes mathematically insufficient, as it collapses a multidimensional interval into a single discrete outcome. In such situations, a probabilistic (Monte Carlo) approach is necessary to estimate the true distribution of compatible CPAK types.

**Table 2:** Summary of the HCT‐9 classification and degree of ambiguity when converting a Hirschmann phenotype to CPAK.

HK‐9 classification: Hirschmann‐CPAK correspondence grid							
HK‐9 cell	Phenotype label	Hirschmann phenotypes	*N* phenotypes Hirschmann	CPAK deterministic	CPAK interval	Ambiguity level	Recommended method
A1	Varus–Varus	VAR3‐15 × VAR3‐15	25	I, IV, VII	I, IV, VII	Low	Deterministic
A2	Neutral–Varus	NEU0 × VAR3‐15	5	I	I, II, IV	Moderate	Stochastic
A3	Valgus–Varus	VAL3‐15 × VAR3‐15	25	I, II, III	I, II, III	Moderate	Deterministic
B1	Varus–Neutral	VAR3‐15 × NEU0	5	IV, VII	I, II, IV, V, VII	High	Stochastic
B2	Neutral–Neutral	NEU0 × NEU0	1	II	I, II, III, IV, V, VI	Maximal	Stochastic
B3	Valgus‐Neutral	VAL3‐15 × NEU0	5	III	II, III, V, VI	High	Stochastic
C1	Varus–Valgus	VAR3‐15 × VAL3‐15	25	IV, V, VI, VII, VIII, IX	IV, V, VI, VII, VIII, IX	Very high	Stochastic
C2	Neutral–Valgus	NEU0 × VAL3‐15	5	VI, IX	III, V, VI, IX	High	Stochastic
C3	Valgus–Valgus	VAL3‐15 × VAL3‐15	25	III, VI, IX	III, VI, IX	Moderate	Deterministic

*Note*: Numbers 3–15 correspond to 3° increments around the mean values (FMA 93°, TMA 87°). *CPAK det*. = CPAK types obtained using the deterministic method (central value). *CPAK interv*. = CPAK types compatible with the full interval (±1.5°).

*Degree of ambiguity*: Number of CPAK types compatible using the interval‐based method. Low = 1–2 types; Moderate = 3–4 types; High = 5 types or more. Cell B2 (NEU0 × NEU0) shows the highest ambiguity with 6 possible CPAK types, justifying the mandatory use of the stochastic method.

*Recommended method*: Deterministic for low‐ambiguity cells (A1, A3); stochastic (Monte Carlo) for high‐ambiguity cells (B1, B2, B3, C1, C2); either approach is acceptable for moderate‐ambiguity cells (A2, C3).

*Clinical note*: Cells B2 (Hirschmann reference) and C1 (discordant varus–valgus configurations) carry the highest risk of CPAK misclassification if Hirschmann's functional classification is used alone without consideration of angular intervals.

Abbreviations: CPAK, Coronal Plane Alignment of the Knee; FMA, femoral mechanical angle; HCT‐9, Hirschmann–CPAK Translational grid; NEU, neutral; TMA, total knee arthroplasty; VAL, valgus; VAR, varus.

## DISCUSSION

### Principal findings

This study demonstrates that there is no one‐to‐one correspondence between Hirschmann FKP and the CPAK classification, the latter being more reductive yet more clinically accessible. When the angular intervals defining each functional phenotype are considered, a single phenotype corresponds on average to 1.7 CPAK types, with a maximum observed in the central B2 cell of the HCT‐9 grid. Deterministic conversion based solely on mean values underestimates this variability. Significant differences between derived distributions and those of the original CPAK cohort were observed with deterministic methods, particularly in NO subjects. In contrast, in the OA population, stochastic conversion no longer demonstrated significant differences, suggesting convergence when intra‐phenotype variability is considered. The HCT‐9 grid identifies areas of high ambiguity in central phenotypes and more stable correspondences at the extremes.

### Geometric and biomechanical interpretation

The first direct comparisons between the CPAK classification and Hirschmann FKP were reported by Jenny and Baldairon [14] and Liu et al. [15]. These studies demonstrated either a limited correlation between the two systems or a superior descriptive capacity of functional phenotypes in specific populations. However, these analyses remained descriptive and population‐based, and none of these analyses formalized the geometric relationship between the two classifications. These findings arise directly from the intrinsic structure of the two systems: functional phenotypes are defined by continuous angular intervals [11] within the broader context of questioning mechanical neutrality [8], whereas CPAK is based on a discrete nine‐category grid defined by fixed thresholds [16]. The substantial variability of HKA, FMA and TMA reported in systematic reviews [6, 17] and confirmed by three‐dimensional CT studies [10] explains why an identical HKA may correspond to different underlying bony configurations. The present mapping demonstrates that this heterogeneity becomes critical when the FMA–TMA interval crosses CPAK thresholds. The maximal ambiguity observed in the B2 cell of the HCT‐9 grid reflects this geometric instability. This finding is consistent with Jenny and Baldairon [14], who concluded that the two systems are complementary rather than interchangeable. Recent studies further confirm that CPAK simplifies the morphological diversity captured by functional phenotypes [2, 12] and does not reproduce certain sagittal variations, such as posterior tibial slope [3]. The convergence observed in the OA population under stochastic conversion suggests a geometric centring effect, whereby FMA–TMA pairs occupy a more restricted region of the bidimensional space. This observation may be related to data showing comparable joint line orientations between OA and NO knees [7] as well as to the hypothesis of selective evolution toward dominant wear patterns [4, 5]. Population‐based studies [13, 15, 19, 21] have similarly reported comparable global trends without establishing a reproducible rule‐based correspondence between functional phenotypes and CPAK types. Moreover, sex‐related differences [1] and potential interactions with sagittal or rotational parameters [20] underscore that coronal alignment represents only one dimension of a more complex three‐dimensional system. Although CPAK provides a pragmatic clinical language, supported by postoperative functional outcome data [18], it does not aim to reproduce the full morphological granularity described by Hirschmann. Therefore, the information loss observed when converting functional phenotypes into CPAK types does not represent a conceptual weakness of CPAK. Rather, it reflects the inevitable mathematical consequence of discretizing continuous anatomical variability. The two classifications are related, but no simple one‐to‐one mapping can exist between them. Regarding the distributions obtained after converting functional phenotypes into CPAK types using the four methodological approaches applied in the present study, comparison with the studies by Jenny and Baldairon [14] and Liu et al. [15] provides additional insight. The discrepancies reported by Jenny et al., particularly the lack of strong correlation between measured HKA and aHKA and the non‐interchangeability of the two classifications, are consistent with the deterministic conversion results reported in the present study. Conversely, the predominance of CPAK Types I–IV observed by Liu et al. in a Chinese OA population can be reproduced when stochastic conversion is applied. By incorporating angular dispersion within functional phenotypes, this model yields a CPAK distribution comparable to that reported in the literature. This convergence in the OA population under a stochastic model suggests a geometric centring effect that had not been captured in previous analyses. The relationship between CPAK and FKP is neither linear nor deterministic but depends on how a continuous anatomical space is projected onto a discrete categorical grid.

### Clinical and practical implications

The relevance of the HCT‐9 grid is grounded in three major observations derived from the present results. First, the presence of a ‘cross‐pattern’ demonstrates that ambiguity in the conversion between functional phenotypes and CPAK types is not random. The most stable correspondences occur at the varus–varus and valgus–valgus extremes, whereas central configurations exhibit the greatest instability. Second, the disappearance of differences in the OA population under the stochastic model indicates that the discordance observed with deterministic methods arises from excessive simplification. In practical terms, this suggests that rigid reliance on a single mean value may lead to imprecise conversion. The apparent instability of CPAK Type V reflects both its central position at the intersection of aHKA and JLO thresholds and the multiplicity of underlying femorotibial combinations capable of producing near‐neutral alignment. Thus, the observed heterogeneity is not solely a statistical artefact of narrow intervals but also a consequence of the intrinsic geometric variability of neutral configurations. HCT‐9 does not constitute a new classification but rather a translational framework that identifies situations in which deterministic conversion is reliable and those in which a probabilistic approach is preferable.

### Strengths and limitations

This study presents several methodological strengths. The mapping between functional phenotypes and CPAK is based on a strictly analytical rather than empirical derivation, ensuring mathematical transparency, reproducibility and theoretical generalizability. Furthermore, the analysis relies on large combined cohorts derived from two landmark studies, enhancing the robustness of the findings at a population level. Finally, the multi‐method approach (deterministic, interval‐based, stochastic and ‘rare’) enables exploration of both point‐based conversion and its probabilistic dimension, providing a comprehensive view of how a continuous anatomical space is projected onto a discrete classification system.

Several limitations must nevertheless be acknowledged. The analysis is based on aggregated data rather than individual FMA and TMA measurements, precluding precise modelling of the true distribution of values and limiting direct comparison between uniform and Gaussian assumptions in Monte Carlo simulations. Furthermore, sex‐ and race‐related variability in FMA and TMA would lead to different CPAK conversion outcomes in populations with demographic profiles distinct from the Hirschmann reference cohort and cannot be modelled without individual‐level data. Methodological differences (supine CT imaging vs. weight‐bearing radiographs), the absence of simultaneous Hirschmann and CPAK measurements in the same patients, and geographically and temporally distinct cohorts may introduce unquantifiable bias. In addition, the HCT‐9 grid is restricted to the coronal plane, does not incorporate sagittal or rotational parameters, assumes a negligible JLCA and has not yet been validated in an independent cohort. Prospective external validation, ideally based on shared individual‐level data, is therefore warranted.

## CONCLUSION

This study demonstrates that the relationship between FKP and the CPAK classification is inherently probabilistic. The loss of information observed during conversion does not reflect a conceptual weakness of the CPAK system, but rather the mathematical consequence of simplifying multidimensional femorotibial variability into a pragmatic nine‐category grid. The HCT‐9 framework provides a structured translational map between the two systems, specifying when deterministic conversion is geometrically reliable and when a probabilistic approach is required, and lays the groundwork for prospective validation using individual‐level clinical data.

## AUTHOR CONTRIBUTIONS

Bernard de Geofroy conceived the study, performed the analytical modelling and statistical analysis and drafted the manuscript. Romain Léonard contributed to data interpretation and manuscript revision. Sebastien Lustig contributed to conceptual development and critical revision of the manuscript. Grégoire Micicoi contributed to the geometric modelling framework and methodological supervision. All authors read and approved the final manuscript.

## FUNDING

The authors have no funding to report.

## CONFLICT OF INTEREST STATEMENT

Sebastien Lustig reports royalties from Stryker, Smith & Nephew and Cerf; consulting fees and honoraria from Stryker; and support for meeting attendance from Stryker. He also holds leadership roles in the European Knee Society and the French Hip and Knee Society. The remaining authors declare no conflict of interest.

## ETHICS STATEMENT

This study is based exclusively on previously published aggregated data and does not involve new patient recruitment or identifiable patient information. Therefore, institutional review board approval was not required. This study did not involve direct patient participation or identifiable patient data.

## Supporting information

Appendix 1: Correspondence between functional knee phenotypes and CPAK classification based on mean mechanical axis, femoral and tibial alignment, and joint line orientation.

Appendix 2: Interval‐based correspondence between functional knee phenotypes and CPAK types derived from femorotibial alignment, mechanical axis (aHKA), and joint line orientation ranges.

Appendix 3: Hirschmann to CPAK Conversion Table (Deterministic and Interval method). Direct lookup table for converting functional knee phenotypes to CPAK types.

## Data Availability

The data that support the findings of this study are derived from previously published datasets (Hirschmann et al. and MacDessi et al.). As the present analysis is based on aggregated data reported in the literature, no new individual‐level patient data were generated. Further details are available from the corresponding author upon reasonable request.
