# Supplementary material for: Speaking the same language? A direct cartography between functional knee phenotypes and CPAK
Source: J Exp Orthop. 2026 Jun 17;13(2):e70801. doi: 10.1002/jeo2.70801 (PMC13274546; doi:10.1002/jeo2.70801)
Supplement: Supplementary file 1 — Appendix 1: Correspondence between functional knee phenotypes and CPAK classification based on mean mechanical axis, femoral and tibial alignment, and joint line orientation. [file JEO2-13-e70801-s001.docx]

Appendix 1: Correspondence between functional knee phenotypes and CPAK classification based on mean mechanical axis, femoral and tibial alignment, and joint line orientation

| **Functional Knee Phenotype** | | | |  | | **CPAK** | | |
| --- | --- | --- | --- | --- | --- | --- | --- | --- |
| **FMA label** | **TMA label** | **FMA center (°)** | **TMA center (°)** | **LDFA (°)** | **MPTA (°)** | **aHKA(°)** | **JLO(°)** | **CPAK type** |
| VAR15 | VAR15 | 78 | 72 | 102 | 72 | -30 | 174 | I |
| VAR12 | VAR15 | 81 | 72 | 99 | 72 | -27 | 171 | I |
| VAR9 | VAR15 | 84 | 72 | 96 | 72 | -24 | 168 | I |
| VAR6 | VAR15 | 87 | 72 | 93 | 72 | -21 | 165 | I |
| VAR3 | VAR15 | 90 | 72 | 90 | 72 | -18 | 162 | I |
| NEU0 | VAR15 | 93 | 72 | 87 | 72 | -15 | 159 | I |
| VAL3 | VAR15 | 96 | 72 | 84 | 72 | -12 | 156 | I |
| VAL6 | VAR15 | 99 | 72 | 81 | 72 | -9 | 153 | I |
| VAL9 | VAR15 | 102 | 72 | 78 | 72 | -6 | 150 | I |
| VAL12 | VAR15 | 105 | 72 | 75 | 72 | -3 | 147 | I |
| VAL15 | VAR15 | 108 | 72 | 72 | 72 | 0 | 144 | II |
| VAR15 | VAR12 | 78 | 75 | 102 | 75 | -27 | 177 | IV |
| VAR12 | VAR12 | 81 | 75 | 99 | 75 | -24 | 174 | I |
| VAR9 | VAR12 | 84 | 75 | 96 | 75 | -21 | 171 | I |
| VAR6 | VAR12 | 87 | 75 | 93 | 75 | -18 | 168 | I |
| VAR3 | VAR12 | 90 | 75 | 90 | 75 | -15 | 165 | I |
| NEU0 | VAR12 | 93 | 75 | 87 | 75 | -12 | 162 | I |
| VAL3 | VAR12 | 96 | 75 | 84 | 75 | -9 | 159 | I |
| VAL6 | VAR12 | 99 | 75 | 81 | 75 | -6 | 156 | I |
| VAL9 | VAR12 | 102 | 75 | 78 | 75 | -3 | 153 | I |
| VAL12 | VAR12 | 105 | 75 | 75 | 75 | 0 | 150 | II |
| VAL15 | VAR12 | 108 | 75 | 72 | 75 | 3 | 147 | III |
| VAR15 | VAR9 | 78 | 78 | 102 | 78 | -24 | 180 | IV |
| VAR12 | VAR9 | 81 | 78 | 99 | 78 | -21 | 177 | IV |
| VAR9 | VAR9 | 84 | 78 | 96 | 78 | -18 | 174 | I |
| VAR6 | VAR9 | 87 | 78 | 93 | 78 | -15 | 171 | I |
| VAR3 | VAR9 | 90 | 78 | 90 | 78 | -12 | 168 | I |
| NEU0 | VAR9 | 93 | 78 | 87 | 78 | -9 | 165 | I |
| VAL3 | VAR9 | 96 | 78 | 84 | 78 | -6 | 162 | I |
| VAL6 | VAR9 | 99 | 78 | 81 | 78 | -3 | 159 | I |
| VAL9 | VAR9 | 102 | 78 | 78 | 78 | 0 | 156 | II |
| VAL12 | VAR9 | 105 | 78 | 75 | 78 | 3 | 153 | III |
| VAL15 | VAR9 | 108 | 78 | 72 | 78 | 6 | 150 | III |
| VAR15 | VAR6 | 78 | 81 | 102 | 81 | -21 | 183 | IV |
| VAR12 | VAR6 | 81 | 81 | 99 | 81 | -18 | 180 | IV |
| VAR9 | VAR6 | 84 | 81 | 96 | 81 | -15 | 177 | IV |
| VAR6 | VAR6 | 87 | 81 | 93 | 81 | -12 | 174 | I |
| VAR3 | VAR6 | 90 | 81 | 90 | 81 | -9 | 171 | I |
| NEU0 | VAR6 | 93 | 81 | 87 | 81 | -6 | 168 | I |
| VAL3 | VAR6 | 96 | 81 | 84 | 81 | -3 | 165 | I |
| VAL6 | VAR6 | 99 | 81 | 81 | 81 | 0 | 162 | II |
| VAL9 | VAR6 | 102 | 81 | 78 | 81 | 3 | 159 | III |
| VAL12 | VAR6 | 105 | 81 | 75 | 81 | 6 | 156 | III |
| VAL15 | VAR6 | 108 | 81 | 72 | 81 | 9 | 153 | III |
| VAR15 | VAR3 | 78 | 84 | 102 | 84 | -18 | 186 | VII |
| VAR12 | VAR3 | 81 | 84 | 99 | 84 | -15 | 183 | IV |
| VAR9 | VAR3 | 84 | 84 | 96 | 84 | -12 | 180 | IV |
| VAR6 | VAR3 | 87 | 84 | 93 | 84 | -9 | 177 | IV |
| VAR3 | VAR3 | 90 | 84 | 90 | 84 | -6 | 174 | I |
| NEU0 | VAR3 | 93 | 84 | 87 | 84 | -3 | 171 | I |
| VAL3 | VAR3 | 96 | 84 | 84 | 84 | 0 | 168 | II |
| VAL6 | VAR3 | 99 | 84 | 81 | 84 | 3 | 165 | III |
| VAL9 | VAR3 | 102 | 84 | 78 | 84 | 6 | 162 | III |
| VAL12 | VAR3 | 105 | 84 | 75 | 84 | 9 | 159 | III |
| VAL15 | VAR3 | 108 | 84 | 72 | 84 | 12 | 156 | III |
| VAR15 | NEU0 | 78 | 87 | 102 | 87 | -15 | 189 | VII |
| VAR12 | NEU0 | 81 | 87 | 99 | 87 | -12 | 186 | VII |
| VAR9 | NEU0 | 84 | 87 | 96 | 87 | -9 | 183 | IV |
| VAR6 | NEU0 | 87 | 87 | 93 | 87 | -6 | 180 | IV |
| VAR3 | NEU0 | 90 | 87 | 90 | 87 | -3 | 177 | IV |
| **NEU0** | **NEU0** | **93** | **87** | **87** | **87** | **0** | **174** | **II** |
| VAL3 | NEU0 | 96 | 87 | 84 | 87 | 3 | 171 | III |
| VAL6 | NEU0 | 99 | 87 | 81 | 87 | 6 | 168 | III |
| VAL9 | NEU0 | 102 | 87 | 78 | 87 | 9 | 165 | III |
| VAL12 | NEU0 | 105 | 87 | 75 | 87 | 12 | 162 | III |
| VAL15 | NEU0 | 108 | 87 | 72 | 87 | 15 | 159 | III |
| VAR15 | VAL3 | 78 | 90 | 102 | 90 | -12 | 192 | VII |
| VAR12 | VAL3 | 81 | 90 | 99 | 90 | -9 | 189 | VII |
| VAR9 | VAL3 | 84 | 90 | 96 | 90 | -6 | 186 | VII |
| VAR6 | VAL3 | 87 | 90 | 93 | 90 | -3 | 183 | IV |
| VAR3 | VAL3 | 90 | 90 | 90 | 90 | 0 | 180 | V |
| NEU0 | VAL3 | 93 | 90 | 87 | 90 | 3 | 177 | VI |
| VAL3 | VAL3 | 96 | 90 | 84 | 90 | 6 | 174 | III |
| VAL6 | VAL3 | 99 | 90 | 81 | 90 | 9 | 171 | III |
| VAL9 | VAL3 | 102 | 90 | 78 | 90 | 12 | 168 | III |
| VAL12 | VAL3 | 105 | 90 | 75 | 90 | 15 | 165 | III |
| VAL15 | VAL3 | 108 | 90 | 72 | 90 | 18 | 162 | III |
| VAR15 | VAL6 | 78 | 93 | 102 | 93 | -9 | 195 | VII |
| VAR12 | VAL6 | 81 | 93 | 99 | 93 | -6 | 192 | VII |
| VAR9 | VAL6 | 84 | 93 | 96 | 93 | -3 | 189 | VII |
| VAR6 | VAL6 | 87 | 93 | 93 | 93 | 0 | 186 | VIII |
| VAR3 | VAL6 | 90 | 93 | 90 | 93 | 3 | 183 | VI |
| NEU0 | VAL6 | 93 | 93 | 87 | 93 | 6 | 180 | VI |
| VAL3 | VAL6 | 96 | 93 | 84 | 93 | 9 | 177 | VI |
| VAL6 | VAL6 | 99 | 93 | 81 | 93 | 12 | 174 | III |
| VAL9 | VAL6 | 102 | 93 | 78 | 93 | 15 | 171 | III |
| VAL12 | VAL6 | 105 | 93 | 75 | 93 | 18 | 168 | III |
| VAL15 | VAL6 | 108 | 93 | 72 | 93 | 21 | 165 | III |
| VAR15 | VAL9 | 78 | 96 | 102 | 96 | -6 | 198 | VII |
| VAR12 | VAL9 | 81 | 96 | 99 | 96 | -3 | 195 | VII |
| VAR9 | VAL9 | 84 | 96 | 96 | 96 | 0 | 192 | VIII |
| VAR6 | VAL9 | 87 | 96 | 93 | 96 | 3 | 189 | IX |
| VAR3 | VAL9 | 90 | 96 | 90 | 96 | 6 | 186 | IX |
| NEU0 | VAL9 | 93 | 96 | 87 | 96 | 9 | 183 | VI |
| VAL3 | VAL9 | 96 | 96 | 84 | 96 | 12 | 180 | VI |
| VAL6 | VAL9 | 99 | 96 | 81 | 96 | 15 | 177 | VI |
| VAL9 | VAL9 | 102 | 96 | 78 | 96 | 18 | 174 | III |
| VAL12 | VAL9 | 105 | 96 | 75 | 96 | 21 | 171 | III |
| VAL15 | VAL9 | 108 | 96 | 72 | 96 | 24 | 168 | III |
| VAR15 | VAL12 | 78 | 99 | 102 | 99 | -3 | 201 | VII |
| VAR12 | VAL12 | 81 | 99 | 99 | 99 | 0 | 198 | VIII |
| VAR9 | VAL12 | 84 | 99 | 96 | 99 | 3 | 195 | IX |
| VAR6 | VAL12 | 87 | 99 | 93 | 99 | 6 | 192 | IX |
| VAR3 | VAL12 | 90 | 99 | 90 | 99 | 9 | 189 | IX |
| NEU0 | VAL12 | 93 | 99 | 87 | 99 | 12 | 186 | IX |
| VAL3 | VAL12 | 96 | 99 | 84 | 99 | 15 | 183 | VI |
| VAL6 | VAL12 | 99 | 99 | 81 | 99 | 18 | 180 | VI |
| VAL9 | VAL12 | 102 | 99 | 78 | 99 | 21 | 177 | VI |
| VAL12 | VAL12 | 105 | 99 | 75 | 99 | 24 | 174 | III |
| VAL15 | VAL12 | 108 | 99 | 72 | 99 | 27 | 171 | III |
| VAR15 | VAL15 | 78 | 102 | 102 | 102 | 0 | 204 | VIII |
| VAR12 | VAL15 | 81 | 102 | 99 | 102 | 3 | 201 | IX |
| VAR9 | VAL15 | 84 | 102 | 96 | 102 | 6 | 198 | IX |
| VAR6 | VAL15 | 87 | 102 | 93 | 102 | 9 | 195 | IX |
| VAR3 | VAL15 | 90 | 102 | 90 | 102 | 12 | 192 | IX |
| NEU0 | VAL15 | 93 | 102 | 87 | 102 | 15 | 189 | IX |
| VAL3 | VAL15 | 96 | 102 | 84 | 102 | 18 | 186 | IX |
| VAL6 | VAL15 | 99 | 102 | 81 | 102 | 21 | 183 | VI |
| VAL9 | VAL15 | 102 | 102 | 78 | 102 | 24 | 180 | VI |
| VAL12 | VAL15 | 105 | 102 | 75 | 102 | 27 | 177 | VI |
| VAL15 | VAL15 | 108 | 102 | 72 | 102 | 30 | 174 | III |
