# Supplementary material for: Speaking the same language? A direct cartography between functional knee phenotypes and CPAK
Source: J Exp Orthop. 2026 Jun 17;13(2):e70801. doi: 10.1002/jeo2.70801 (PMC13274546; doi:10.1002/jeo2.70801)
Supplement: Supplementary file 2 — Appendix 2: Interval‐based correspondence between functional knee phenotypes and CPAK types derived from femorotibial alignment, mechanical axis (aHKA), and joint line orientation ranges. [file JEO2-13-e70801-s003.docx]

Appendix 2: Interval-based correspondence between functional knee phenotypes and CPAK types derived from femorotibial alignment, mechanical axis (aHKA), and joint line orientation ranges

| **Functional Knee Phenotype** | | | | | |  | | | | **CPAK** | | | | |
| --- | --- | --- | --- | --- | --- | --- | --- | --- | --- | --- | --- | --- | --- | --- |
| **FMA label** | **TMA label** | **FMA min** | **FMA max** | **TMA min** | **TMA max** | **LDFA min** | **LDFA max** | **MPTA min** | **MPTA max** | **aHKA min** | **aHKA max** | **JLO min** | **JLO max** | **CPAK possible** |
| VAR15 | VAR15 | 76,5 | 79,5 | 70,5 | 73,5 | 100,5 | 103,5 | 70,5 | 73,5 | -33 | -27 | 171 | 177 | I,IV |
| VAR12 | VAR15 | 79,5 | 82,5 | 70,5 | 73,5 | 97,5 | 100,5 | 70,5 | 73,5 | -30 | -24 | 168 | 174 | I |
| VAR9 | VAR15 | 82,5 | 85,5 | 70,5 | 73,5 | 94,5 | 97,5 | 70,5 | 73,5 | -27 | -21 | 165 | 171 | I |
| VAR6 | VAR15 | 85,5 | 88,5 | 70,5 | 73,5 | 91,5 | 94,5 | 70,5 | 73,5 | -24 | -18 | 162 | 168 | I |
| VAR3 | VAR15 | 88,5 | 91,5 | 70,5 | 73,5 | 88,5 | 91,5 | 70,5 | 73,5 | -21 | -15 | 159 | 165 | I |
| NEU0 | VAR15 | 91,5 | 94,5 | 70,5 | 73,5 | 85,5 | 88,5 | 70,5 | 73,5 | -18 | -12 | 156 | 162 | I |
| VAL3 | VAR15 | 94,5 | 97,5 | 70,5 | 73,5 | 82,5 | 85,5 | 70,5 | 73,5 | -15 | -9 | 153 | 159 | I |
| VAL6 | VAR15 | 97,5 | 100,5 | 70,5 | 73,5 | 79,5 | 82,5 | 70,5 | 73,5 | -12 | -6 | 150 | 156 | I |
| VAL9 | VAR15 | 100,5 | 103,5 | 70,5 | 73,5 | 76,5 | 79,5 | 70,5 | 73,5 | -9 | -3 | 147 | 153 | I |
| VAL12 | VAR15 | 103,5 | 106,5 | 70,5 | 73,5 | 73,5 | 76,5 | 70,5 | 73,5 | -6 | 0 | 144 | 150 | I,II |
| VAL15 | VAR15 | 106,5 | 109,5 | 70,5 | 73,5 | 70,5 | 73,5 | 70,5 | 73,5 | -3 | 3 | 141 | 147 | I,II,III |
| VAR15 | VAR12 | 76,5 | 79,5 | 73,5 | 76,5 | 100,5 | 103,5 | 73,5 | 76,5 | -30 | -24 | 174 | 180 | I,IV |
| VAR12 | VAR12 | 79,5 | 82,5 | 73,5 | 76,5 | 97,5 | 100,5 | 73,5 | 76,5 | -27 | -21 | 171 | 177 | I,IV |
| VAR9 | VAR12 | 82,5 | 85,5 | 73,5 | 76,5 | 94,5 | 97,5 | 73,5 | 76,5 | -24 | -18 | 168 | 174 | I |
| VAR6 | VAR12 | 85,5 | 88,5 | 73,5 | 76,5 | 91,5 | 94,5 | 73,5 | 76,5 | -21 | -15 | 165 | 171 | I |
| VAR3 | VAR12 | 88,5 | 91,5 | 73,5 | 76,5 | 88,5 | 91,5 | 73,5 | 76,5 | -18 | -12 | 162 | 168 | I |
| NEU0 | VAR12 | 91,5 | 94,5 | 73,5 | 76,5 | 85,5 | 88,5 | 73,5 | 76,5 | -15 | -9 | 159 | 165 | I |
| VAL3 | VAR12 | 94,5 | 97,5 | 73,5 | 76,5 | 82,5 | 85,5 | 73,5 | 76,5 | -12 | -6 | 156 | 162 | I |
| VAL6 | VAR12 | 97,5 | 100,5 | 73,5 | 76,5 | 79,5 | 82,5 | 73,5 | 76,5 | -9 | -3 | 153 | 159 | I |
| VAL9 | VAR12 | 100,5 | 103,5 | 73,5 | 76,5 | 76,5 | 79,5 | 73,5 | 76,5 | -6 | 0 | 150 | 156 | I,II |
| VAL12 | VAR12 | 103,5 | 106,5 | 73,5 | 76,5 | 73,5 | 76,5 | 73,5 | 76,5 | -3 | 3 | 147 | 153 | I,II,III |
| VAL15 | VAR12 | 106,5 | 109,5 | 73,5 | 76,5 | 70,5 | 73,5 | 73,5 | 76,5 | 0 | 6 | 144 | 150 | II,III |
| VAR15 | VAR9 | 76,5 | 79,5 | 76,5 | 79,5 | 100,5 | 103,5 | 76,5 | 79,5 | -27 | -21 | 177 | 183 | IV |
| VAR12 | VAR9 | 79,5 | 82,5 | 76,5 | 79,5 | 97,5 | 100,5 | 76,5 | 79,5 | -24 | -18 | 174 | 180 | I,IV |
| VAR9 | VAR9 | 82,5 | 85,5 | 76,5 | 79,5 | 94,5 | 97,5 | 76,5 | 79,5 | -21 | -15 | 171 | 177 | I,IV |
| VAR6 | VAR9 | 85,5 | 88,5 | 76,5 | 79,5 | 91,5 | 94,5 | 76,5 | 79,5 | -18 | -12 | 168 | 174 | I |
| VAR3 | VAR9 | 88,5 | 91,5 | 76,5 | 79,5 | 88,5 | 91,5 | 76,5 | 79,5 | -15 | -9 | 165 | 171 | I |
| NEU0 | VAR9 | 91,5 | 94,5 | 76,5 | 79,5 | 85,5 | 88,5 | 76,5 | 79,5 | -12 | -6 | 162 | 168 | I |
| VAL3 | VAR9 | 94,5 | 97,5 | 76,5 | 79,5 | 82,5 | 85,5 | 76,5 | 79,5 | -9 | -3 | 159 | 165 | I |
| VAL6 | VAR9 | 97,5 | 100,5 | 76,5 | 79,5 | 79,5 | 82,5 | 76,5 | 79,5 | -6 | 0 | 156 | 162 | I,II |
| VAL9 | VAR9 | 100,5 | 103,5 | 76,5 | 79,5 | 76,5 | 79,5 | 76,5 | 79,5 | -3 | 3 | 153 | 159 | I,II,III |
| VAL12 | VAR9 | 103,5 | 106,5 | 76,5 | 79,5 | 73,5 | 76,5 | 76,5 | 79,5 | 0 | 6 | 150 | 156 | II,III |
| VAL15 | VAR9 | 106,5 | 109,5 | 76,5 | 79,5 | 70,5 | 73,5 | 76,5 | 79,5 | 3 | 9 | 147 | 153 | III |
| VAR15 | VAR6 | 76,5 | 79,5 | 79,5 | 82,5 | 100,5 | 103,5 | 79,5 | 82,5 | -24 | -18 | 180 | 186 | IV,VII |
| VAR12 | VAR6 | 79,5 | 82,5 | 79,5 | 82,5 | 97,5 | 100,5 | 79,5 | 82,5 | -21 | -15 | 177 | 183 | IV |
| VAR9 | VAR6 | 82,5 | 85,5 | 79,5 | 82,5 | 94,5 | 97,5 | 79,5 | 82,5 | -18 | -12 | 174 | 180 | I,IV |
| VAR6 | VAR6 | 85,5 | 88,5 | 79,5 | 82,5 | 91,5 | 94,5 | 79,5 | 82,5 | -15 | -9 | 171 | 177 | I,IV |
| VAR3 | VAR6 | 88,5 | 91,5 | 79,5 | 82,5 | 88,5 | 91,5 | 79,5 | 82,5 | -12 | -6 | 168 | 174 | I |
| NEU0 | VAR6 | 91,5 | 94,5 | 79,5 | 82,5 | 85,5 | 88,5 | 79,5 | 82,5 | -9 | -3 | 165 | 171 | I |
| VAL3 | VAR6 | 94,5 | 97,5 | 79,5 | 82,5 | 82,5 | 85,5 | 79,5 | 82,5 | -6 | 0 | 162 | 168 | I,II |
| VAL6 | VAR6 | 97,5 | 100,5 | 79,5 | 82,5 | 79,5 | 82,5 | 79,5 | 82,5 | -3 | 3 | 159 | 165 | I,II,III |
| VAL9 | VAR6 | 100,5 | 103,5 | 79,5 | 82,5 | 76,5 | 79,5 | 79,5 | 82,5 | 0 | 6 | 156 | 162 | II,III |
| VAL12 | VAR6 | 103,5 | 106,5 | 79,5 | 82,5 | 73,5 | 76,5 | 79,5 | 82,5 | 3 | 9 | 153 | 159 | III |
| VAL15 | VAR6 | 106,5 | 109,5 | 79,5 | 82,5 | 70,5 | 73,5 | 79,5 | 82,5 | 6 | 12 | 150 | 156 | III |
| VAR15 | VAR3 | 76,5 | 79,5 | 82,5 | 85,5 | 100,5 | 103,5 | 82,5 | 85,5 | -21 | -15 | 183 | 189 | IV,VII |
| VAR12 | VAR3 | 79,5 | 82,5 | 82,5 | 85,5 | 97,5 | 100,5 | 82,5 | 85,5 | -18 | -12 | 180 | 186 | IV,VII |
| VAR9 | VAR3 | 82,5 | 85,5 | 82,5 | 85,5 | 94,5 | 97,5 | 82,5 | 85,5 | -15 | -9 | 177 | 183 | IV |
| VAR6 | VAR3 | 85,5 | 88,5 | 82,5 | 85,5 | 91,5 | 94,5 | 82,5 | 85,5 | -12 | -6 | 174 | 180 | I,IV |
| VAR3 | VAR3 | 88,5 | 91,5 | 82,5 | 85,5 | 88,5 | 91,5 | 82,5 | 85,5 | -9 | -3 | 171 | 177 | I,IV |
| NEU0 | VAR3 | 91,5 | 94,5 | 82,5 | 85,5 | 85,5 | 88,5 | 82,5 | 85,5 | -6 | 0 | 168 | 174 | I,II |
| VAL3 | VAR3 | 94,5 | 97,5 | 82,5 | 85,5 | 82,5 | 85,5 | 82,5 | 85,5 | -3 | 3 | 165 | 171 | I,II,III |
| VAL6 | VAR3 | 97,5 | 100,5 | 82,5 | 85,5 | 79,5 | 82,5 | 82,5 | 85,5 | 0 | 6 | 162 | 168 | II,III |
| VAL9 | VAR3 | 100,5 | 103,5 | 82,5 | 85,5 | 76,5 | 79,5 | 82,5 | 85,5 | 3 | 9 | 159 | 165 | III |
| VAL12 | VAR3 | 103,5 | 106,5 | 82,5 | 85,5 | 73,5 | 76,5 | 82,5 | 85,5 | 6 | 12 | 156 | 162 | III |
| VAL15 | VAR3 | 106,5 | 109,5 | 82,5 | 85,5 | 70,5 | 73,5 | 82,5 | 85,5 | 9 | 15 | 153 | 159 | III |
| VAR15 | NEU0 | 76,5 | 79,5 | 85,5 | 88,5 | 100,5 | 103,5 | 85,5 | 88,5 | -18 | -12 | 186 | 192 | VII |
| VAR12 | NEU0 | 79,5 | 82,5 | 85,5 | 88,5 | 97,5 | 100,5 | 85,5 | 88,5 | -15 | -9 | 183 | 189 | IV,VII |
| VAR9 | NEU0 | 82,5 | 85,5 | 85,5 | 88,5 | 94,5 | 97,5 | 85,5 | 88,5 | -12 | -6 | 180 | 186 | IV,VII |
| VAR6 | NEU0 | 85,5 | 88,5 | 85,5 | 88,5 | 91,5 | 94,5 | 85,5 | 88,5 | -9 | -3 | 177 | 183 | IV |
| VAR3 | NEU0 | 88,5 | 91,5 | 85,5 | 88,5 | 88,5 | 91,5 | 85,5 | 88,5 | -6 | 0 | 174 | 180 | I,II,IV,V |
| **NEU0** | **NEU0** | **91,5** | **94,5** | **85,5** | **88,5** | **85,5** | **88,5** | **85,5** | **88,5** | **-3** | **3** | **171** | **177** | **I,II,III,IV,V,VI** |
| VAL3 | NEU0 | 94,5 | 97,5 | 85,5 | 88,5 | 82,5 | 85,5 | 85,5 | 88,5 | 0 | 6 | 168 | 174 | II,III |
| VAL6 | NEU0 | 97,5 | 100,5 | 85,5 | 88,5 | 79,5 | 82,5 | 85,5 | 88,5 | 3 | 9 | 165 | 171 | III |
| VAL9 | NEU0 | 100,5 | 103,5 | 85,5 | 88,5 | 76,5 | 79,5 | 85,5 | 88,5 | 6 | 12 | 162 | 168 | III |
| VAL12 | NEU0 | 103,5 | 106,5 | 85,5 | 88,5 | 73,5 | 76,5 | 85,5 | 88,5 | 9 | 15 | 159 | 165 | III |
| VAL15 | NEU0 | 106,5 | 109,5 | 85,5 | 88,5 | 70,5 | 73,5 | 85,5 | 88,5 | 12 | 18 | 156 | 162 | III |
| VAR15 | VAL3 | 76,5 | 79,5 | 88,5 | 91,5 | 100,5 | 103,5 | 88,5 | 91,5 | -15 | -9 | 189 | 195 | VII |
| VAR12 | VAL3 | 79,5 | 82,5 | 88,5 | 91,5 | 97,5 | 100,5 | 88,5 | 91,5 | -12 | -6 | 186 | 192 | VII |
| VAR9 | VAL3 | 82,5 | 85,5 | 88,5 | 91,5 | 94,5 | 97,5 | 88,5 | 91,5 | -9 | -3 | 183 | 189 | IV,VII |
| VAR6 | VAL3 | 85,5 | 88,5 | 88,5 | 91,5 | 91,5 | 94,5 | 88,5 | 91,5 | -6 | 0 | 180 | 186 | IV,V,VII,VIII |
| VAR3 | VAL3 | 88,5 | 91,5 | 88,5 | 91,5 | 88,5 | 91,5 | 88,5 | 91,5 | -3 | 3 | 177 | 183 | IV,V,VI |
| NEU0 | VAL3 | 91,5 | 94,5 | 88,5 | 91,5 | 85,5 | 88,5 | 88,5 | 91,5 | 0 | 6 | 174 | 180 | II,III,V,VI |
| VAL3 | VAL3 | 94,5 | 97,5 | 88,5 | 91,5 | 82,5 | 85,5 | 88,5 | 91,5 | 3 | 9 | 171 | 177 | III,VI |
| VAL6 | VAL3 | 97,5 | 100,5 | 88,5 | 91,5 | 79,5 | 82,5 | 88,5 | 91,5 | 6 | 12 | 168 | 174 | III |
| VAL9 | VAL3 | 100,5 | 103,5 | 88,5 | 91,5 | 76,5 | 79,5 | 88,5 | 91,5 | 9 | 15 | 165 | 171 | III |
| VAL12 | VAL3 | 103,5 | 106,5 | 88,5 | 91,5 | 73,5 | 76,5 | 88,5 | 91,5 | 12 | 18 | 162 | 168 | III |
| VAL15 | VAL3 | 106,5 | 109,5 | 88,5 | 91,5 | 70,5 | 73,5 | 88,5 | 91,5 | 15 | 21 | 159 | 165 | III |
| VAR15 | VAL6 | 76,5 | 79,5 | 91,5 | 94,5 | 100,5 | 103,5 | 91,5 | 94,5 | -12 | -6 | 192 | 198 | VII |
| VAR12 | VAL6 | 79,5 | 82,5 | 91,5 | 94,5 | 97,5 | 100,5 | 91,5 | 94,5 | -9 | -3 | 189 | 195 | VII |
| VAR9 | VAL6 | 82,5 | 85,5 | 91,5 | 94,5 | 94,5 | 97,5 | 91,5 | 94,5 | -6 | 0 | 186 | 192 | VII,VIII |
| VAR6 | VAL6 | 85,5 | 88,5 | 91,5 | 94,5 | 91,5 | 94,5 | 91,5 | 94,5 | -3 | 3 | 183 | 189 | IV,V,VI,VII,VIII,IX |
| VAR3 | VAL6 | 88,5 | 91,5 | 91,5 | 94,5 | 88,5 | 91,5 | 91,5 | 94,5 | 0 | 6 | 180 | 186 | V,VI,VIII,IX |
| NEU0 | VAL6 | 91,5 | 94,5 | 91,5 | 94,5 | 85,5 | 88,5 | 91,5 | 94,5 | 3 | 9 | 177 | 183 | VI |
| VAL3 | VAL6 | 94,5 | 97,5 | 91,5 | 94,5 | 82,5 | 85,5 | 91,5 | 94,5 | 6 | 12 | 174 | 180 | III,VI |
| VAL6 | VAL6 | 97,5 | 100,5 | 91,5 | 94,5 | 79,5 | 82,5 | 91,5 | 94,5 | 9 | 15 | 171 | 177 | III,VI |
| VAL9 | VAL6 | 100,5 | 103,5 | 91,5 | 94,5 | 76,5 | 79,5 | 91,5 | 94,5 | 12 | 18 | 168 | 174 | III |
| VAL12 | VAL6 | 103,5 | 106,5 | 91,5 | 94,5 | 73,5 | 76,5 | 91,5 | 94,5 | 15 | 21 | 165 | 171 | III |
| VAL15 | VAL6 | 106,5 | 109,5 | 91,5 | 94,5 | 70,5 | 73,5 | 91,5 | 94,5 | 18 | 24 | 162 | 168 | III |
| VAR15 | VAL9 | 76,5 | 79,5 | 94,5 | 97,5 | 100,5 | 103,5 | 94,5 | 97,5 | -9 | -3 | 195 | 201 | VII |
| VAR12 | VAL9 | 79,5 | 82,5 | 94,5 | 97,5 | 97,5 | 100,5 | 94,5 | 97,5 | -6 | 0 | 192 | 198 | VII,VIII |
| VAR9 | VAL9 | 82,5 | 85,5 | 94,5 | 97,5 | 94,5 | 97,5 | 94,5 | 97,5 | -3 | 3 | 189 | 195 | VII,VIII,IX |
| VAR6 | VAL9 | 85,5 | 88,5 | 94,5 | 97,5 | 91,5 | 94,5 | 94,5 | 97,5 | 0 | 6 | 186 | 192 | VIII,IX |
| VAR3 | VAL9 | 88,5 | 91,5 | 94,5 | 97,5 | 88,5 | 91,5 | 94,5 | 97,5 | 3 | 9 | 183 | 189 | VI,IX |
| NEU0 | VAL9 | 91,5 | 94,5 | 94,5 | 97,5 | 85,5 | 88,5 | 94,5 | 97,5 | 6 | 12 | 180 | 186 | VI,IX |
| VAL3 | VAL9 | 94,5 | 97,5 | 94,5 | 97,5 | 82,5 | 85,5 | 94,5 | 97,5 | 9 | 15 | 177 | 183 | VI |
| VAL6 | VAL9 | 97,5 | 100,5 | 94,5 | 97,5 | 79,5 | 82,5 | 94,5 | 97,5 | 12 | 18 | 174 | 180 | III,VI |
| VAL9 | VAL9 | 100,5 | 103,5 | 94,5 | 97,5 | 76,5 | 79,5 | 94,5 | 97,5 | 15 | 21 | 171 | 177 | III,VI |
| VAL12 | VAL9 | 103,5 | 106,5 | 94,5 | 97,5 | 73,5 | 76,5 | 94,5 | 97,5 | 18 | 24 | 168 | 174 | III |
| VAL15 | VAL9 | 106,5 | 109,5 | 94,5 | 97,5 | 70,5 | 73,5 | 94,5 | 97,5 | 21 | 27 | 165 | 171 | III |
| VAR15 | VAL12 | 76,5 | 79,5 | 97,5 | 100,5 | 100,5 | 103,5 | 97,5 | 100,5 | -6 | 0 | 198 | 204 | VII,VIII |
| VAR12 | VAL12 | 79,5 | 82,5 | 97,5 | 100,5 | 97,5 | 100,5 | 97,5 | 100,5 | -3 | 3 | 195 | 201 | VII,VIII,IX |
| VAR9 | VAL12 | 82,5 | 85,5 | 97,5 | 100,5 | 94,5 | 97,5 | 97,5 | 100,5 | 0 | 6 | 192 | 198 | VIII,IX |
| VAR6 | VAL12 | 85,5 | 88,5 | 97,5 | 100,5 | 91,5 | 94,5 | 97,5 | 100,5 | 3 | 9 | 189 | 195 | IX |
| VAR3 | VAL12 | 88,5 | 91,5 | 97,5 | 100,5 | 88,5 | 91,5 | 97,5 | 100,5 | 6 | 12 | 186 | 192 | IX |
| NEU0 | VAL12 | 91,5 | 94,5 | 97,5 | 100,5 | 85,5 | 88,5 | 97,5 | 100,5 | 9 | 15 | 183 | 189 | VI,IX |
| VAL3 | VAL12 | 94,5 | 97,5 | 97,5 | 100,5 | 82,5 | 85,5 | 97,5 | 100,5 | 12 | 18 | 180 | 186 | VI,IX |
| VAL6 | VAL12 | 97,5 | 100,5 | 97,5 | 100,5 | 79,5 | 82,5 | 97,5 | 100,5 | 15 | 21 | 177 | 183 | VI |
| VAL9 | VAL12 | 100,5 | 103,5 | 97,5 | 100,5 | 76,5 | 79,5 | 97,5 | 100,5 | 18 | 24 | 174 | 180 | III,VI |
| VAL12 | VAL12 | 103,5 | 106,5 | 97,5 | 100,5 | 73,5 | 76,5 | 97,5 | 100,5 | 21 | 27 | 171 | 177 | III,VI |
| VAL15 | VAL12 | 106,5 | 109,5 | 97,5 | 100,5 | 70,5 | 73,5 | 97,5 | 100,5 | 24 | 30 | 168 | 174 | III |
| VAR15 | VAL15 | 76,5 | 79,5 | 100,5 | 103,5 | 100,5 | 103,5 | 100,5 | 103,5 | -3 | 3 | 201 | 207 | VII,VIII,IX |
| VAR12 | VAL15 | 79,5 | 82,5 | 100,5 | 103,5 | 97,5 | 100,5 | 100,5 | 103,5 | 0 | 6 | 198 | 204 | VIII,IX |
| VAR9 | VAL15 | 82,5 | 85,5 | 100,5 | 103,5 | 94,5 | 97,5 | 100,5 | 103,5 | 3 | 9 | 195 | 201 | IX |
| VAR6 | VAL15 | 85,5 | 88,5 | 100,5 | 103,5 | 91,5 | 94,5 | 100,5 | 103,5 | 6 | 12 | 192 | 198 | IX |
| VAR3 | VAL15 | 88,5 | 91,5 | 100,5 | 103,5 | 88,5 | 91,5 | 100,5 | 103,5 | 9 | 15 | 189 | 195 | IX |
| NEU0 | VAL15 | 91,5 | 94,5 | 100,5 | 103,5 | 85,5 | 88,5 | 100,5 | 103,5 | 12 | 18 | 186 | 192 | IX |
| VAL3 | VAL15 | 94,5 | 97,5 | 100,5 | 103,5 | 82,5 | 85,5 | 100,5 | 103,5 | 15 | 21 | 183 | 189 | VI,IX |
| VAL6 | VAL15 | 97,5 | 100,5 | 100,5 | 103,5 | 79,5 | 82,5 | 100,5 | 103,5 | 18 | 24 | 180 | 186 | VI,IX |
| VAL9 | VAL15 | 100,5 | 103,5 | 100,5 | 103,5 | 76,5 | 79,5 | 100,5 | 103,5 | 21 | 27 | 177 | 183 | VI |
| VAL12 | VAL15 | 103,5 | 106,5 | 100,5 | 103,5 | 73,5 | 76,5 | 100,5 | 103,5 | 24 | 30 | 174 | 180 | III,VI |
| VAL15 | VAL15 | 106,5 | 109,5 | 100,5 | 103,5 | 70,5 | 73,5 | 100,5 | 103,5 | 27 | 33 | 171 | 177 | III,VI |
