# Supplementary material for: Speaking the same language? A direct cartography between functional knee phenotypes and CPAK
Source: J Exp Orthop. 2026 Jun 17;13(2):e70801. doi: 10.1002/jeo2.70801 (PMC13274546; doi:10.1002/jeo2.70801)
Supplement: Supplementary file 3 — Appendix 3: Hirschmann to CPAK Conversion Table (Deterministic and Interval method). Direct lookup table for converting functional knee phenotypes to CPAK types. [file JEO2-13-e70801-s002.docx]

Appendix 3: Hirschmann to CPAK Conversion Table (Deterministic and Interval method). *Direct lookup table for converting functional knee phenotypes to CPAK types*

How to use this table: (1) Identify the Hirschmann phenotype (e.g., VAR6×NEU0), (2) Read corresponding CPAK type(s). CPAK Deterministic = type from central values. CPAK Interval = all compatible types within ±1.5° range.

| Hirschmann Phenotype | FMA (°) | TMA (°) | CPAK Deterministic | CPAK Interval |
| --- | --- | --- | --- | --- |
| VAR15×VAR15 | 78 | 72 | I | I,IV |
| VAR12×VAR15 | 81 | 72 | I | I |
| VAR9×VAR15 | 84 | 72 | I | I |
| VAR6×VAR15 | 87 | 72 | I | I |
| VAR3×VAR15 | 90 | 72 | I | I |
| NEU0×VAR15 | 93 | 72 | I | I |
| VAL3×VAR15 | 96 | 72 | I | I |
| VAL6×VAR15 | 99 | 72 | I | I |
| VAL9×VAR15 | 102 | 72 | I | I |
| VAL12×VAR15 | 105 | 72 | I | I,II |
| VAL15×VAR15 | 108 | 72 | II | I,II,III |
| VAR15×VAR12 | 78 | 75 | IV | I,IV |
| VAR12×VAR12 | 81 | 75 | I | I,IV |
| VAR9×VAR12 | 84 | 75 | I | I |
| VAR6×VAR12 | 87 | 75 | I | I |
| VAR3×VAR12 | 90 | 75 | I | I |
| NEU0×VAR12 | 93 | 75 | I | I |
| VAL3×VAR12 | 96 | 75 | I | I |
| VAL6×VAR12 | 99 | 75 | I | I |
| VAL9×VAR12 | 102 | 75 | I | I,II |
| VAL12×VAR12 | 105 | 75 | II | I,II,III |
| VAL15×VAR12 | 108 | 75 | III | II,III |
| VAR15×VAR9 | 78 | 78 | IV | IV |
| VAR12×VAR9 | 81 | 78 | IV | I,IV |
| VAR9×VAR9 | 84 | 78 | I | I,IV |
| VAR6×VAR9 | 87 | 78 | I | I |
| VAR3×VAR9 | 90 | 78 | I | I |
| NEU0×VAR9 | 93 | 78 | I | I |
| VAL3×VAR9 | 96 | 78 | I | I |
| VAL6×VAR9 | 99 | 78 | I | I,II |
| VAL9×VAR9 | 102 | 78 | II | I,II,III |
| VAL12×VAR9 | 105 | 78 | III | II,III |
| VAL15×VAR9 | 108 | 78 | III | III |
| VAR15×VAR6 | 78 | 81 | IV | IV,VII |
| VAR12×VAR6 | 81 | 81 | IV | IV |
| VAR9×VAR6 | 84 | 81 | IV | I,IV |
| VAR6×VAR6 | 87 | 81 | I | I,IV |
| VAR3×VAR6 | 90 | 81 | I | I |
| NEU0×VAR6 | 93 | 81 | I | I |
| VAL3×VAR6 | 96 | 81 | I | I,II |
| VAL6×VAR6 | 99 | 81 | II | I,II,III |
| VAL9×VAR6 | 102 | 81 | III | II,III |
| VAL12×VAR6 | 105 | 81 | III | III |
| VAL15×VAR6 | 108 | 81 | III | III |
| VAR15×VAR3 | 78 | 84 | VII | IV,VII |
| VAR12×VAR3 | 81 | 84 | IV | IV,VII |
| VAR9×VAR3 | 84 | 84 | IV | IV |
| VAR6×VAR3 | 87 | 84 | IV | I,IV |
| VAR3×VAR3 | 90 | 84 | I | I,IV |
| NEU0×VAR3 | 93 | 84 | I | I,II |
| VAL3×VAR3 | 96 | 84 | II | I,II,III |
| VAL6×VAR3 | 99 | 84 | III | II,III |
| VAL9×VAR3 | 102 | 84 | III | III |
| VAL12×VAR3 | 105 | 84 | III | III |
| VAL15×VAR3 | 108 | 84 | III | III |
| VAR15×NEU0 | 78 | 87 | VII | VII |
| VAR12×NEU0 | 81 | 87 | VII | IV,VII |
| VAR9×NEU0 | 84 | 87 | IV | IV,VII |
| VAR6×NEU0 | 87 | 87 | IV | IV |
| VAR3×NEU0 | 90 | 87 | IV | I,II,IV,V |
| NEU0×NEU0 | 93 | 87 | II | I,II,III,IV,V,VI |
| VAL3×NEU0 | 96 | 87 | III | II,III |
| VAL6×NEU0 | 99 | 87 | III | III |
| VAL9×NEU0 | 102 | 87 | III | III |
| VAL12×NEU0 | 105 | 87 | III | III |
| VAL15×NEU0 | 108 | 87 | III | III |
| VAR15×VAL3 | 78 | 90 | VII | VII |
| VAR12×VAL3 | 81 | 90 | VII | VII |
| VAR9×VAL3 | 84 | 90 | VII | IV,VII |
| VAR6×VAL3 | 87 | 90 | IV | IV,V,VII,VIII |
| VAR3×VAL3 | 90 | 90 | V | IV,V,VI |
| NEU0×VAL3 | 93 | 90 | VI | II,III,V,VI |
| VAL3×VAL3 | 96 | 90 | III | III,VI |
| VAL6×VAL3 | 99 | 90 | III | III |
| VAL9×VAL3 | 102 | 90 | III | III |
| VAL12×VAL3 | 105 | 90 | III | III |
| VAL15×VAL3 | 108 | 90 | III | III |
| VAR15×VAL6 | 78 | 93 | VII | VII |
| VAR12×VAL6 | 81 | 93 | VII | VII |
| VAR9×VAL6 | 84 | 93 | VII | VII,VIII |
| VAR6×VAL6 | 87 | 93 | VIII | IV,V,VI,VII,VIII,IX |
| VAR3×VAL6 | 90 | 93 | VI | V,VI,VIII,IX |
| NEU0×VAL6 | 93 | 93 | VI | VI |
| VAL3×VAL6 | 96 | 93 | VI | III,VI |
| VAL6×VAL6 | 99 | 93 | III | III,VI |
| VAL9×VAL6 | 102 | 93 | III | III |
| VAL12×VAL6 | 105 | 93 | III | III |
| VAL15×VAL6 | 108 | 93 | III | III |
| VAR15×VAL9 | 78 | 96 | VII | VII |
| VAR12×VAL9 | 81 | 96 | VII | VII,VIII |
| VAR9×VAL9 | 84 | 96 | VIII | VII,VIII,IX |
| VAR6×VAL9 | 87 | 96 | IX | VIII,IX |
| VAR3×VAL9 | 90 | 96 | IX | VI,IX |
| NEU0×VAL9 | 93 | 96 | VI | VI,IX |
| VAL3×VAL9 | 96 | 96 | VI | VI |
| VAL6×VAL9 | 99 | 96 | VI | III,VI |
| VAL9×VAL9 | 102 | 96 | III | III,VI |
| VAL12×VAL9 | 105 | 96 | III | III |
| VAL15×VAL9 | 108 | 96 | III | III |
| VAR15×VAL12 | 78 | 99 | VII | VII,VIII |
| VAR12×VAL12 | 81 | 99 | VIII | VII,VIII,IX |
| VAR9×VAL12 | 84 | 99 | IX | VIII,IX |
| VAR6×VAL12 | 87 | 99 | IX | IX |
| VAR3×VAL12 | 90 | 99 | IX | IX |
| NEU0×VAL12 | 93 | 99 | IX | VI,IX |
| VAL3×VAL12 | 96 | 99 | VI | VI,IX |
| VAL6×VAL12 | 99 | 99 | VI | VI |
| VAL9×VAL12 | 102 | 99 | VI | III,VI |
| VAL12×VAL12 | 105 | 99 | III | III,VI |
| VAL15×VAL12 | 108 | 99 | III | III |
| VAR15×VAL15 | 78 | 102 | VIII | VII,VIII,IX |
| VAR12×VAL15 | 81 | 102 | IX | VIII,IX |
| VAR9×VAL15 | 84 | 102 | IX | IX |
| VAR6×VAL15 | 87 | 102 | IX | IX |
| VAR3×VAL15 | 90 | 102 | IX | IX |
| NEU0×VAL15 | 93 | 102 | IX | IX |
| VAL3×VAL15 | 96 | 102 | IX | VI,IX |
| VAL6×VAL15 | 99 | 102 | VI | VI,IX |
| VAL9×VAL15 | 102 | 102 | VI | VI |
| VAL12×VAL15 | 105 | 102 | VI | III,VI |
| VAL15×VAL15 | 108 | 102 | III | III,VI |

**Note:** This conversion table lists all 121 Hirschmann functional knee phenotypes with their corresponding CPAK types. **For clinical use:** If only one CPAK type is listed under "Interval", use deterministic conversion. If multiple types are listed, use stochastic (Monte Carlo) method for population-level attribution.
